# Transcriptome profiling of longissimus dorsi during different prenatal stages to identify genes involved in intramuscular fat deposition in lean and obese pig breeds

**DOI:** 10.1007/s11033-023-09088-8

**Published:** 2024-03-05

**Authors:** Jun Feng Chen, Jing Wang, Jin Chai, Wei Jin, Qiao Ling Ren, Qiang Ma, Qing Xia Lu, Jia Jie Sun, De Lin Mo, Jia Qing Zhang, Bao Song Xing

**Affiliations:** 1 Henan Key Laboratory of Farm Animal Breeding and Nutritional Regulation, Institute of Animal Husbandry and Veterinary Science, Henan Academy of Agricultural Sciences, Huayuan Road No.116, Zhengzhou, 450002 Henan China; 2 Agricultural Ministry Key Laboratory of Swine Breeding and Genetics & Key Laboratory of Agricultural Animal Genetics, Breeding, and Reproduction of Ministry of Education, Huazhong Agricultural University, Wuhan, 430070 China; 3 Guangdong Provincial Key Lab of Agro-Animal Genomics and Molecular Breeding, National Engineering Research Center for Breeding Swine Industry, College of Animal Science, South China Agricultural University, Guangzhou, Guangdong China; 4 State Key Laboratory of Biocontrol, School of Life Sciences, Sun Yat-Sen University, Guangzhou, Guangdong China

**Keywords:** Transcriptome, Muscle development, Meat quality trait, Pig, Different embryonic stages

## Abstract

**Background:**

There was significant difference in muscle development between fat-type and lean-type pig breeds.

**Methods and results:**

In current study, transcriptome analysis and bioinformatics analysis were used to compare the difference in longissimus dorsi (LD) muscle at three time-points (38 days post coitus (dpc), 58 dpc, and 78 dpc ) between Huainan (HN) and Large white (LW) pig breeds. A total of 24500 transcripts were obtained in 18 samples, and 2319, 2799, and 3713 differently expressed genes (DEGs) were identified between these two breeds at 38 dpc, 58 dpc, and 78 dpc, respectively. And the number and foldchange of DEGs were increased, the alternative splice also increased. The cluster analysis of DEGs indicated the embryonic development progress of LD muscle between these two breeds was different. There were 539 shared DEGs between HN and LW at three stages, and the top-shared DEGs were associated with muscle development and lipid deposition, such as KLF4, NR4A1, HSP70, ZBTB16 and so on.

**Conclusions:**

The results showed DEGs between Huainan (HN) and Large white (LW) pig breeds, and contributed to the understanding the muscle development difference between HN and LW, and provided basic materials for improvement of meat quality.

**Supplementary Information:**

The online version contains supplementary material available at 10.1007/s11033-023-09088-8.

## Introduction

Pork is the main source of animal protein, especially for Chinese consumers, 60% of animal protein comes from pork [1]. Researching the regulation mechanism of muscle development will not only help to increase meat yield, but also help to improve meat quality. Muscle development is a complicated process, including the formation of muscle fibers in embryonic period, the muscle fibers development after birth, and muscle regeneration in adulthood [2]. The embryonic stage is the most important, the number of muscle fibers is generally fixed at this stage. After birth, muscle development is mainly form new muscle fibers (the proliferation, differentiation, and fusion of skeletal muscle satellite cells) and proliferation of muscle fibers [3]. Meat yield is closely related to the number and volume of muscle fibers [4]. Previous researches indicated that there were two growth waves during prenatal muscle development, the first wave was 35–60 days post coitus (dpc) to form the primary muscle fibers, and the second wave was 54–90 dpc to form the secondary fibers [3, 5]. Compared with Western pig breeds (lean-type), Chinese local pig breeds (fat-type) showed thinner muscle fibers, more intramuscular fat deposition content, redder meat color, but slower growth rate, and higher feed-to-meat ratio [6]. The genetic differences between these two pig breeds provided good research materials for meat quality. It had been reported that, during embryonic stage, primary fibers formed earlier in Lantang (LT, Chinese local pig breeds, more obese) pig, but its second fibers were less and thinner than that in Landrace (LR, more lean) [7]. Compared with Yorkshire (YK, lean-type), Tongcheng (TC, fat-type) pigs had more myoblasts at 30 dpc, and the primary muscle fibers could be found in both breeds at 40 dpc. YK pigs showed the secondary muscle fibers at 55 dpc, but TC had secondary muscle fibers much later [7]. These researches indicated that the appearance time and the formation process of myoblasts, the primary and secondary muscle fibers were different between Chinese indigenous pigs and Western pigs, these differences were closely related to the differences in muscle fiber volume and meat yield between these two breeds. Meanwhile, the embryonic muscle development was associated with intramuscular fat content. Wigmore and Stickland found that, the embryonic adipogenesis was synchronized with the formation of secondary muscle fibers [3]. In production, during late pregnancy improving female animals’ nutritional level could increase the intramuscular fat content of the offspring. These indicated that the embryonic stage has an important influence on muscle fiber development and intramuscular fat deposition. The number and volume of muscle fibers, and intramuscular fat content affect meat quality and yield, so embryonic muscle development is a critical period for studying meat quality traits.

Huainan (HN) pigs, an excellent Chinese indigenous breed, were included in “fine livestock and poultry breeds in Henan province” in 1986 [8]. HN pigs were mainly distributed in the upper reaches of the Huai River, and were famous for its roughage- and heat- resistance, large litter size, high intramuscular fat content, thin muscle fibers [9]. Previously we had researched the effect of castration on development of muscle and adipose in HN pigs [10, 11], until now there hasn’t report about the transcriptomic difference between HN and western pig breeds. In this study, we firstly compared the gene expression profiling of longissimus dorsi muscle between HN and Large White (LW) pigs at 38, 58, and 78 dpc. The results were helpful to understand the differences in molecular regulation mechanism of embryonic muscle development between fat-type and lean-type pig breeds, and provided basic materials for improving meat quality traits.

## Materials and methods

### Materials

Five healthy female HN and LW pigs were obtained from the Henan Xing Rui Agricultural and Animal Husbandry Technology Co., LTD in Henan province, China. All of these sows (in their second or third parity) were raised under the same condition, and they were inseminated with fresh semen from the boars of the same breed. At 38, 58, and 78 dpc, one sow from each breed were randomly selected and killed by sodium pentobarbital (50 mg/kg body weight). Three male and three female fetuses were selected for sample preparation. At 38 dpc, it’s difficult for distinguish the sex of the fetuses, so all of the fetuses were used for sample preparation, and their sex was identified by SRY (sex determining region Y) gene. LD muscle at the same position were quickly dissected and immediately frozen in liquid nitrogen.

### Methods

#### Total RNA isolation, library preparation and Illumina sequencing

According to the manufacturer’s instruction, total RNA was isolated from the LD muscle by using TRIzol reagent (Invitrogen, Carlsbad, USA). Agarose gel electrophoresis, Agilent 2100 Bioanalyzer (Agilent Technologies, Massy, France), and Nano-Drop ND-2000 spectrophotometer (Nano-Drop products, Wilmington, USA) were performed to detect the purity, integrity, and quality of the RNA. Qubit RNA BR was used to assess the concentration of the RNA, only samples with an RNA integrity number (RIN) of greater than eight were used for sequencing.

Ribo-ZeroTM kit (Epicentre, Madison, WI, USA) was used to remove ribosomal RNA (rRNA), and NEBNext Ultra Directional RNA Library Prep Kit for Illumina (New England BioLabs Inc., Beverly, MA, USA) was used to create a sequencing library. For each breed, one female RNA sample and one male RNA sample from the same stage were mixed, so there were three mixed samples for sequencing at each stage. The 18 libraries were sequenced with a HiSeq 2500 platform (125 bp PE, Illumina, San Diego, CA, USA).

#### Reads mapping and transcript assembly

The clean reads were obtained by removing the low-quality sequences and adapter sequences for the raw reads using FASTQC (http://www.bioinformatics.babraham.ac.uk/projects/fastqc/) and the NGS QC TOOLKIT (http://59.163.192.90:8080/ngsqctoolkit/). TopHat2 software was applied to map clean reads to the porcine reference genome (Sscrofa 11.1) with default parameters. Cufflinks was applied to perform the transcript assembly and abundance estimation.

#### SNP and InDel analysis and variable splices prediction

GATK software was used to identify the single nucleotide polymorphism (SNP) site and insertion-deletion (InDel). rMATS software was used to obtained the variable splicing types and corresponding expressions of each sample. And Cufflink was applied to estimate the differentially expressed isoforms.

#### Differentially expressed mRNA identification and pathway analysis

Differentially expressed mRNAs (DEMs) were detected by using DESeq software package with P_adj_ < 0.05 and | log_2_FoldChange | > 0.25. The cluster analysis of DEGs was performed by using “ConensusClusterPlus” software package. DAVID tool was applied to perform Gene Ontology (GO) terms and the Kyoto Encyclopedia of Genes and Genomes (KEGG) analyses of DEMs. Circle plots of GO terms and expression trends of the shared DEGs was performed by using OmicShare tools, a free online platform for data analysis (https://www.omicshare.com/tools). The enriched terms were considered statistically significant when the p value was less than 0.05. The shared DEGs was visualized by using the R package “VennDiagrams”. The heat map of the top-26 shared DEGs was performed by using the heat map package.

#### Validation of DEGs expression in RNA-sequencing by RT-qPCR (reverse transcription real-time quantitative PCR)

To validate DEMs’ expressional trend, RT-qPCR was performed to analyze the expressional levels of four genes, NR4A1 (nuclear hormone receptor nuclear receptor subfamily 4, group a, member 1), KLF4 (kruppel-like factor 4), HSP70.2 (heat-shock protein 70.2), and ZBTB16 (zinc finger and BTB domain containing 16) were selected from the top-26 shared DEGs in Fig. 3b at 78 dpc between HN and LW pigs. The primers for these genes and GAPDH (glyceraldehyde-3-phosphate dehydrogenase) were list in Table 1. The RNA used in RT-qPCR was the same as that used in the RNA-sequencing. PrimeScript® RT reagent Kit (Perfect Real Time) (TaKaRa, Kusatsu, Japan) was used for the qPCR. Each reaction including 2 × SYBR Premix Ex Taq™ 12.5 µL, forward primer (10 µmol/L) 1.0 µL, reverse primer (10 µmol/L) 1.0 µL, cDNA 2 µL and ddH_2_O 8.5 µL. The amplification including an initial denaturation step, 95 ℃ for 40 s, and 40 cycles (95 °C for 5 s and 60 °C for 30 s). Each amplification was performed in triplicate, and the relative expression of mRNAs were calculated using the 2^−△△Ct^ method.

**Table 1:** Primers used in real-time quantitative RT-qPCR

Loci	Primer sequence		Size (bp)
	Forward (5'–3')	Reverse (5'–3')	
NR4A1	GCCCTGTATCCAAGCCCAAT	GGTCACTTGCCAGGTGGTG	72
KLF4	GAGGGAGACGGAGGAGTTCAAT	GGAGGAAGAGGATGAGGCTG	124
HSP70	GACGGAAGCACAGGAAGGA	GAAGACAGGGTGCGTTTGG	91
ZBTB16	GCTGTGGCAAGAAGTTCAGC	TCGAAGGGCTTCTCTCCTGT	76
HIF-1α	TGTACCTTAACTAGCAGGGGGA	ACCCACACTGAGACTGGTTAT	118
GAPDH	CTGCCCCTTCTGCTGATGC	TCCACGATGCCGAAGTTGTC	151

### Statistical analysis

R package was used to analyzed the data, and results were presented as mean ± SE, and it was considered as significant when *p* < 0.05.

## Results

### SNP, InDel, and alternative splice detection

**Fig. 1:**
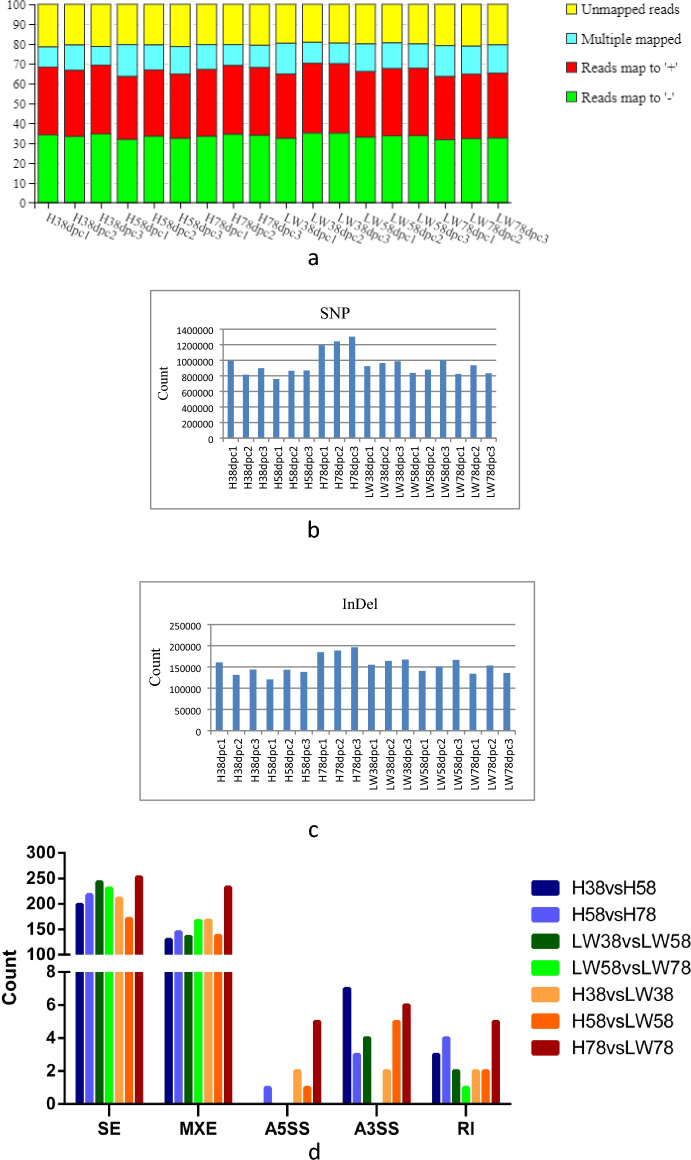
Features of transcript detected in LD muscle of HN and LW at 38, 58, and 78 dpc. **a** Mapping statistics of each sample against the Sus scrofa reference genome 11.1. The numbers of SNPs (**b**) and InDel (**c**) detected in each sample. **d** The number of alternative splice at different comparison. *SE* Skipped exon, *MXE* Mutually exclusive exon, *A5SS* Alternative 5′ splice site, *A3SS* Alternative 3′ splice site, *RI* Retained intron

A total of 18 LD muscle samples from the HN and LW fetus at 38, 58, and 78 dpc were used for transcriptome sequencing. Against the Sus scrofa reference genome 11.1, the ratio of mapped to “+” and “−” strand of each sample was similar (Fig. 1a). There were more SNPs and InDel in 78 dpc of HN (Fig. 1b and c).

Five alternative splicing events, SE (skippedexon), MXE (mutually exclusive exon), A5SS (alternative 5′ splice site), A3SS (alternative 3′ splice site), and RI (retained intron) were found in all of 18 samples (Fig. 1d), and these five alternative splicing events with the same trends in each sample, SE was the highest, and then was MXE, RI was the lowest. Compared the difference of alternative splicing events between HN and LW at 38 dpc, 58 dpc, and 78 dpc, the difference was highest at 78 dpc.

### The DEGs detection

**Fig. 2:**
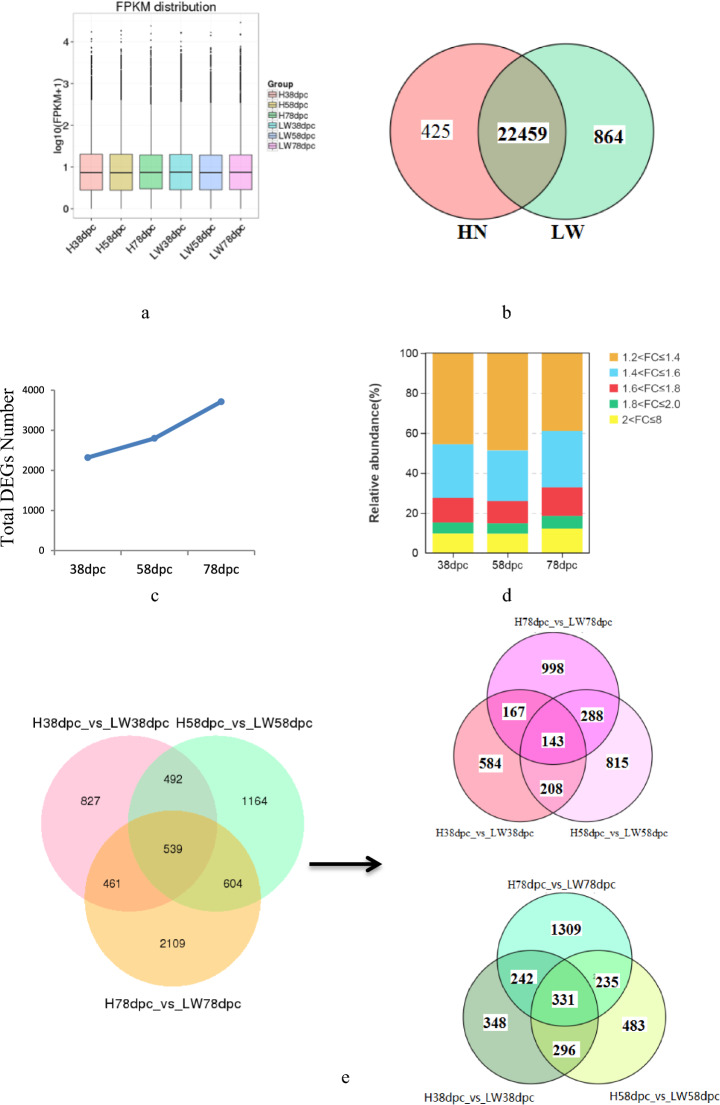
The differently expressed genes (DEGs) in LD muscle between HN and LW pigs. **a** FPKM distribution of genes detected in HN and LW at each time point. **b** Venn map of genes shared by these two breeds. **c** The total number of DEGs between HN and LW breeds at 38, 58, and 78 dpc. **d** Districution of DEGs’ foldchange between these two breeds at 38, 58, and 78 dpc. **e** Venn maps of the number of DEGs (left panel), upregulated DEGs (right panel, red) and downregulated DEGs (right panel, green) between these two breeds at three time-points. (Color figure online)

Approximately 24,500 transcripts were detected in all of samples, and the FPKM (fragments per kilobase of exon model per million mapped fragments) distribution of mRNAs in these six groups was similar (Fig. 2a). There were 22459 shared mRNAs between these two pig breeds, and 425 and 864 unique mRNAs in HN and LW, respectively (Fig. 2b).

**Fig. 3:**
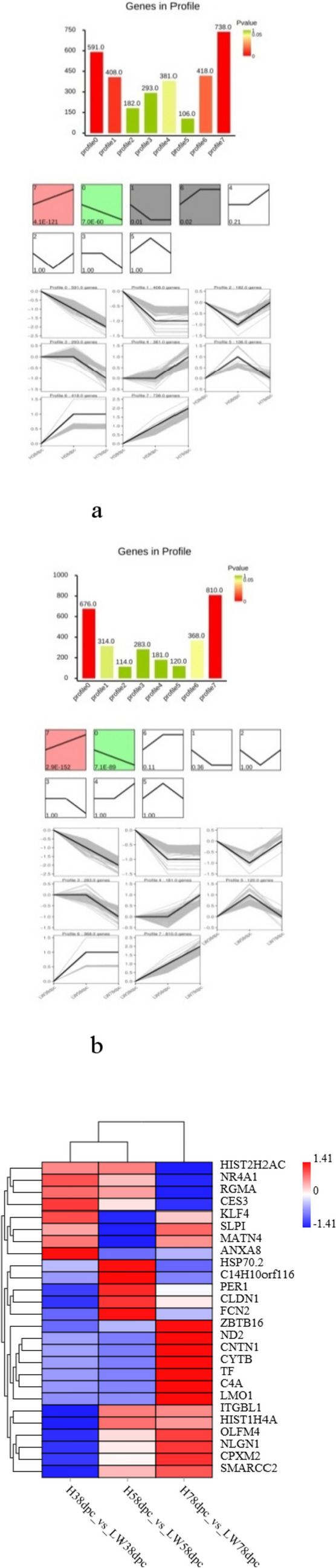
The features of the shared DEGs at 38, 58, and 78 dpc between HN and LW pigs. **a** Expression trends of the shared DEGs in HN. **b** Expression trends of the shared DEGs in LW. **c** Heat map of the top-26 shared DEGs. The color bar showed the foldchange of DEGs. (Color figure online)

As the embryo develops, there were 2319, 2799, and 3713 DEGs between these two breeds at 38dpc, 58 dpc, and 78 dpc, respectively. The number of DEGs between these two breeds was increased (Fig. 2c). The cluster analysis indicated that the conservation of three replicates for each stage was good. At each time point, three samples from same breed were clustered together firstly, and then the samples from different breeds clustered together. The samples from HN at 58 dpc were clustered with the samples from these two breeds at 38 dpc, but the samples from LW at 58 dpc were clustered with the samples from these two breeds at 78 dpc (Supplement Fig. 1). For each stage, the lowly altered DEGs (1.2 < FC < 1.4) accouted for the largest proportion of DEG, but at 78 dpc, the proportion of lowly altered was reduced, suggesting that the difference at 78 dpc was more significantly (Fig. 2d). There were 539 shared DEGs between these two breeds at three stages, of which 143 upregulated and 331 downregulated (Fig. 2e, Supplementary Table 1). The shared 539 DEGs were performed cluster analysis at different stages for HN and LW, respectively. The results indicated that there were 8 expression profiles in both breeds (Fig. 3a and b). The heat map of the top-26 shared DEGs (with｜log_2_FoldChange｜> 0.8 at each stage ) were shown in Fig. 3c.

### GO annotation and KEGG pathway mapping

**Fig. 4:**
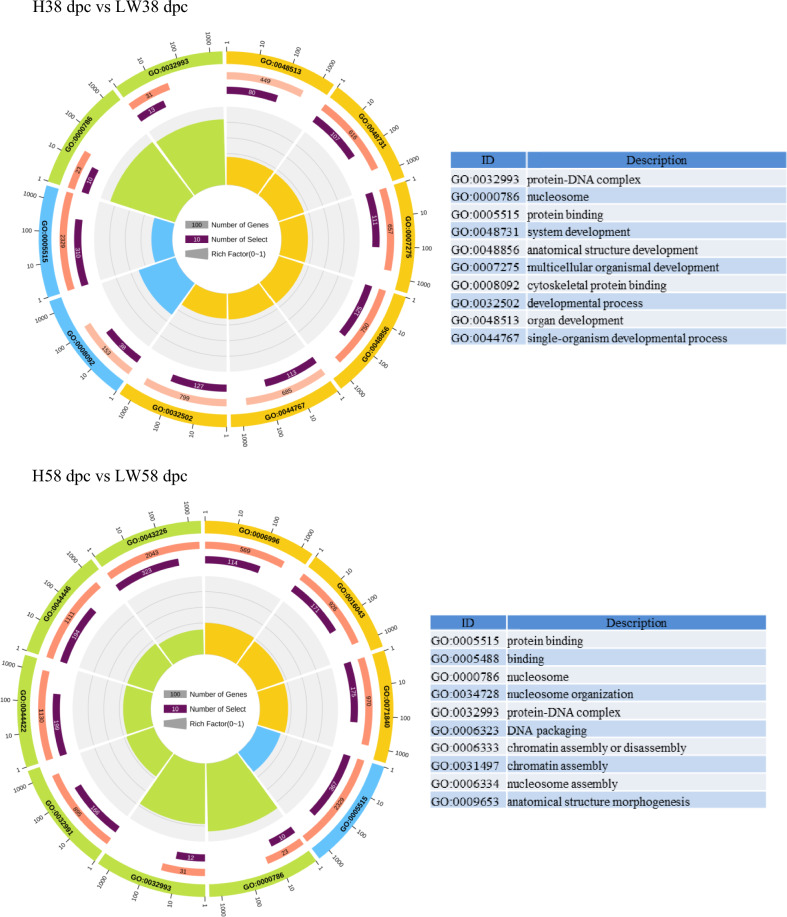

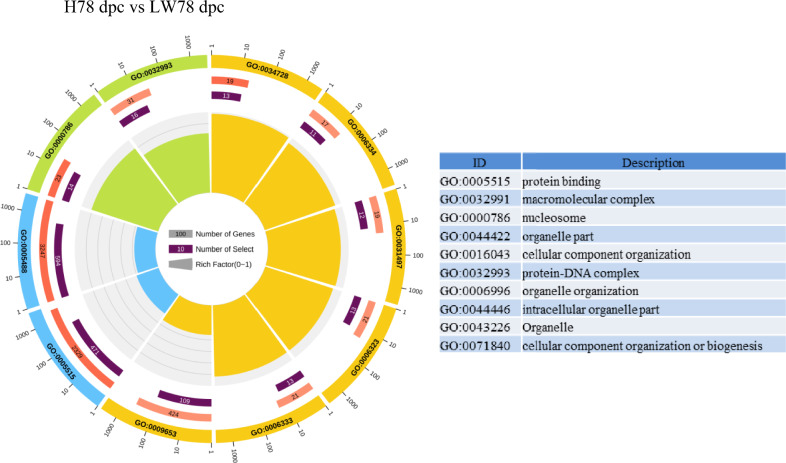
Circle plot of enriched GO pathway between HN and LW at 38, 58, and 78 dpc. The outer circle showed the name of enriched GO pathway, and yellow scale showed biological process, blue scale showed molecular function, and green scale showed cellular component. The number in red bar (the second circle) indicated the number of background genes in this pathway, the more genes, the longer the bar, the smaller Q value, the redder the color. The number in purple bars of (the third circle) indicated the DEGs in this pathway. The fourth circle indicated the RichFactor value of each pathway, and each small grid of the background auxiliary line represented 0.1. (Color figure online)

According to GO enrichment of DEGs between these two breeds (Fig. 4), the protein-DNA complex, nucleosome, and protein binding were enriched for all of three stages. KEGG analysis indicated that (Table 2) calcium, HIF-1 (hypoxia inducible factor-1), insulin, rap1 pathways were continuously higher in HN in comparison to LW. At 38 and 58 dpc, ErbB (erythroblastic leukemia viral oncogene homolog), estrogen, lysine, thyroid hormone, Wnt, PI3K-AKT (phosphatidylinositol-3-kinase), focal adhension pathways were continuously lower in HN than in LW, but at 78 dpc, the difference wasn’t significantly. At 78 dpc, the pathway related with glycolipid metabolism was significantly different between these two breeds, such as fatty acid related pathway, biosynthesis of amino acid, TCA (tricarboxylic acid cycle) cycle, insulin, glyoxylate and dicarboxylated metabolism and so on.

**Table 2:** The signaling pathway enriched at different stages between Huainan (HN) and Large white (LW) pigs

Signaling pathway	HN38 vs. LW38	HN58 vs. LW58	HN78 vs. LW78
Calcium signaling pathway	↑	↑	↑
HIF-1 signaling pathway	↑	↑	↑
Insulin signaling pathway	↑	↑	↑
Rap1 signaling pathway	↑	↑	↑
ARVC	↑	↑	
ErbB signaling pathway	↑	↑	
Estrogen signaling pathway	↑	↑	
Lysine degradation	↑	↑	
Thyroid hormone signaling pathway	↑	↑	
Wnt signaling pathway	↑	↑	
mTOR signaling pathway	↑	↑	
PI3K-Akt signaling pathway	↑	↑	
Focal adhesion	↑	↑	
MAPK signaling pathway		↑	↑
Steroid biosynthesis	↑		↑
ECM-receptor interaction	↑	↑	↓
Glycerolipid metabolism	↑		
Protein digestion and absorption	↑		
Carbonhydrate digestion and absorption		↓	
cGMP-PKG signaling pathway		↓	
TGF-beta signaling pathway	↓		
AMPK signaling pathway		↑	
cAMP signaling pathway		↑	
Adrenergic signaling in cardiomyocytes			↑
Biosynthesis of amino acids			↑
Carbon metabolism			↑
Cardiac muscle contraction			↑
TCA cycle			↑
Fatty acid degradation			↑
Fatty acid elongation			↑
Fatty acid metabolism			↑
Glycolysis/gluconeogenesis			↑
Glyoxylate and dicarboxylated metabolism			↑
HCM			↑
Insulin secretion			↑
Metabolic pathways			↑
NAFLD			↑
Oxidative phosphorylation			↑
Protein processing in endoplasmic reticulum			↑
Pyruvate metabolism			↑
Valine, leucine and isoleucine degradation			↑

For each breed, KEGG analysis results indicated that adrenergic signaling in cardiomyocytes and HIF-1 signaling were continuously upregulated respectively (Table 3), and metabolic pathway were continuously downregulated in both breeds. For both breeds, PI3K-AKT pathway was downregulated at 58 dpc but upregulated at 78 dpc. In HN pigs, calcium, glycolysis/gluconeogenesis, cGMP-PKG (cyclic guanosine monophosphate-protein kinase G) were continuously upregulated, but only regulated at 78 dpc in LW pigs. TCA cycle, NAFLD (nonalcoholic fatty liver disease), MAPK (mitogen activated protein kinase) were upregulated at 58 dpc in HN, but upregulated at 78 dpc in LW. Some pathways showed opposite trends at 58 vs. 38 dpc and 78 vs. 58 dpc, such as PI3K-AKT, WNT, TGF-beta (transforming growth factor beta). Type II diabetes mellitus, mTOR (mechanistic target of rapamycin), insulin, cAMP (cyclicadenosine monophosphate) only upregulated at 78 vs. 58 in HN, glycerolipid metabolism, ErbB (erythroblastic oncogene B), arginine and proline metabolism only downregulated at 78 vs. 58 dpc in LW.

**Table 3:** The signaling pathway enriched at Huainan (HN) and Large white (LW) pigs between different stages

Signaling pathway	HN58 vs. HN38	LW58 vs. LW38	HN78 vs. HN58	LW78 vs. LW58
Adrenergic signaling in cardiomyocytes	↓	↓	↓	↓
HIF-1 signaling pathway	↓	↓	↓	↓
Calcium signaling pathway	↓	↓	↓	↓
Glycolysis/gluconeogenesis	↓	↓	↓	↓
cGMP-PKG signaling pathway	↓	↓	↓	↓
Insulin signaling pathway	↓	↓	↓	↓
Rap1 signaling pathway	↓	↓	↓	↓
ARVC	↓	↓	↓	↓
Focal adhesion	↓	↓	↓	↓
AMPK			↓	↓
HCM			↓	↓
FoxO signaling pathway			↓	↓
TCA cycle	↓			↓
NAFLD	↓			↓
MAPK signaling pathway	↓			↓
Metabolic pathways	↑	↑	↑	↑
Steroid biosynthesis	↑	↑		↑
Lysine degradation	↑	↑		
Biosynthesis of unsaturated fatty acid	↑	↑		
PI3K-Akt signaling pathway	↑	↑	↓	↓
Wnt signaling pathway	↑	↑	↓	
TGF-beta signaling pathway	↑	↑	↓	
Biosynthesis of amino acids	↑	↑		↓
ECM-receptor interaction		↑	↓	↓
Thyroid hormone signaling pathway		↑	↓	
Carbon metabolism		↑		↓
Protein digestion and absorption		↑	↓	↓
Carbonhydrate digestion and absorption		↑	↓	
Propanoate metabolism	↓			↓
Valine, leucine and isoleucine degradation	↓			
Fatty acid degradation	↓			
Type II diabetes mellitus			↓	
mTOR signaling pathway			↓	
Insulin secretion			↓	
cAMP signaling pathway			↓	
Glycerolipid metabolism				↑
ErbB signaling pathway				↑
Arginine and proline metabolism				↑
Cardiac muscle contraction		↓		
Oxidative phosphorylation				↓
Fatty acid elongation		↑		
Fatty acid metabolism		↑		
Protein processing in endoplasmic reticulum		↑		
Fatty acid biosynthesis		↑		

### Validation of the expressional level of DEMs

**Fig. 5:**
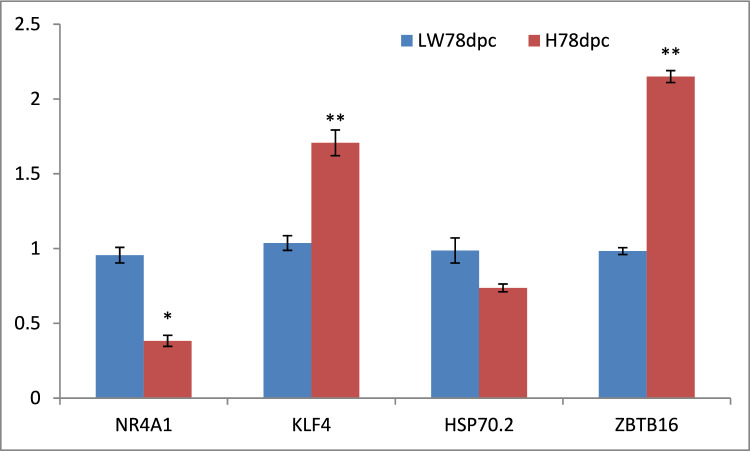
Validation of the expression of the genes by RT-qPCR. The data represented the Mean ± SE, ** indicates *p* < 0.01, * indicates *p* < 0.05

To validate the DEMs’ expressional trends in RNA-sequencing, the expressional level of four top-26 shared DEGs between HN and LW at 78 dpc were detected by RT-qPCR (Fig. 5). The expressional level of KLF4 and ZBTB16 was upregulated in HN, and the expressional level of NR4A1 and HSP70.2 was downregulated in HN.

## Discussions

The molecular regulation mechanism of muscle development is complicated and is not clear so far. We firstly used HN and LW pigs as model animals toperforme the transcriptome sequencing of LD muscle at 38, 58, and 78 dpc.

Five kinds of alternative splice had been detected in different corporations, SE and MXE account for the largest proportion (Fig. 1d). A5SS wasn’t detected in H58 vs. HN38, LW58 vs. LW38, and LW78 vs. LW58. A3SS wasn’t detected in LW78 vs. LW58. In HN pigs, the difference of alternative splice between different stages wasn’t significantly, only more A3SS in HN58 vs. HN38 than that in H78 vs. H58. Comparison of alternative splice in each time point between HN and LW pigs, it’s obvious that there were much more splice in 78 dpc than that in 38 dpc and 58 dpc, indicating that the difference was most significantly in 78 dpc. Similarly, the total number of DEGs between these two breeds was gradually increased during embryo development (Fig. 2c). In 78 dpc, the number of DEGs was about two fold than that of in 38 dpc, and the foldchange of DEGs was higher than that in previous two stages (Fig. 2e).

Previous research showed that the primary muscle fiber was found at 35 dpc in LT, 49 dpc in LR. For the formation of primary muscle fibers, LT pigs showed faster develop speed [7]. Another research compared the embryonic muscle development difference between TC and YK, the results showed that there were much more myoblast in TC at 30 dpc [5]. These results indicated that compared with western pig breeds, myogenic differentiation started earlier and primary muscle fibers formed earlier in Chinese local pigs. In current research, the cluster analysis of DEGs indicated that the different stages of two pig breeds were firstly enriched together, 58 dpc in HN were enriched with 38 dpc, but 58 dpc in LW were enriched with 78 dpc (Fig. 2d). The results revealed that the embryonic development progress of LD muscle between these two breeds was different.

There were 539 shared DEGs between these two breeds at three stages, the top differentially expressed genes were shown in Fig. 3b, many of them were associated with muscle development and lipid deposition. For example, KLF4 expressed early during adipogenesis and its expression trend was similar with C/EBP beta. C/EBP beta was activated by KLF4, leading to activation of transcriptional cascade in adipogenesis. Meanwhile C/EBP beta regulated KLF4 through a negative feedback loop [12]. Ubiquitination of KLF4 was necessary for adipogenesis of 3T3-L1 precursor cells [13]. The inhibition of A2b adenosine receptor on murine adipogenesis was relay on KLF4 [14]. Cholecalciferol [15], GSK3 beta (glycogen synthase kinase-3) [16], miR-25 [17] participated in regulation of KLF4.

NR4A1, regulated glycolysis and glucose transport in liver, muscle, and adipose tissue through different signaling pathway [18–20]. The expression of NR4A1 was downregulated in diabetic, ob/ob, and db/db mice. Compared with wild mice, NR4A1 knockout mice were more likely obesity [19]. In skeletal muscle, activated by compounds or sport, NR4A1 could promoted glycogen synthesis and participated in insulin resistance [21, 22]. NR4A1 could inhibit adipogenesis and lipid deposition in primary adipocytes, meanwhile NR4A subfamily could regulate lipid metabolism [23]. It was reported that HSP70 was related with tenderness of bovine LD muscle [24], the content of HSP70 in longissimus thoracis and semitendinosus of steer was higher than that in bull [25]. The SNPs in ZBTB16 were associated with body mass index, waist to hip ratio, and LDL cholesterol levels [26]. In rat, losing one copy of Zbtb16 caused reduced body weight and adiposity [27].

HIF-1α enhanced myogenesis by inducing WNT7A, the latter one was essential for muscle differentiation [28]. In current research, HIF-1α was continuously upregulated in both of two breeds. In the comparison between HN and LW, HIF-1α was continuously higher in HN than in LW. Thyroid hormone inhibited muscle satellite cells proliferation [29], promoted myogenic differentiation [30], promoted the expression of fast myosin heavy chain and regulated the conversation of slow-fast muscle fibers [31]. Thyroid hormone also participated in skeletal muscle mitochondrial biosynthesis and enhanced skeletal muscle metabolism, thermogenesis and glucose intake [32, 33]. In this study, thyroid pathway was higher at 38 and 58 dpc in HN than in LW. MAPK participated in promoting myoblast proliferation, and its expression level was highest at 38 dpc and 58 dpc in HN and LW, respectively. These results coincided with the earlier myogenic differentiation in Chinese indigenous pig breed but longer myoblast proliferation in western pig breeds.

Evidences showed that PI3K could promote adipogenesis [34], and its expression was much higher in HN than that in LW at 38 dpc and 58 dpc. This result coincided with higher IMF (intramuscular fat) content in Chinese local pigs.

It was reported that Wnt pathway play a critical role in regulation of muscle fiber type formation, Wnt5a enhanced proliferation of slow muscle fiber, Wnt11 promotes fast muscle fiber formation [35]. Wnt pathway showed higher expressional level in HN than in LW at 38 dpc and 58 dpc. At 38 dpc, Wnt5a showed higher expressional level in HN than in LW. In HN pigs, Wnt pathway was firstly upregulated at 38 dpc and then downregulated at 78 dpc, but in LW pigs, its expression was only upregulated in 58 dpc. Calcium pathway, which could promotes slow muscle fiber formation, was continuously higher in HN than in LW in all stages. These results coincided with the thinner muscle fibers volume in HN.

## Conclusion

In general, the transcriptome profiling of LD muscle at different embryonic stages between HN and LW was compared, the results indicated that genes participated in the regulation of differences between HN and LW at embryonic muscle development by regulating proliferation, myogenesis, adipogenesis and muscle fiber transformation. HIF-1α, thyroid、MAPK, PI3K, Wnt pathways might be used as pivotal pathway for research of meat quality.

## Supplementary Information

Below is the link to the electronic supplementary material.

Supplementary material 1 (DOCX 61.6 kb)

Supplementary material 2 (XLSX 118.3 kb)

## Data Availability

The raw data generated for this study had been submitted to NCBI Sequence Read Archive with accession number SRP243554.

## Abbreviations

LD: Longissimus dorsi
HN: Huainan
LW: Large white
DEGs: Differently expressed genes
IMF: Intramuscular fat
dpc: Days post coitus
LT: Lantang
LR: Landrace
YK: Yorkshire
TC: Tongcheng
SRY: Sex determining region Y
RIN: RNA integrity number
rRNA: Ribosomal RNA
SNP: Single nucleotide polymorphism
InDel: Insertion-deletion
DEMs: Differentially expressed mRNAs
Go: Gene Ontology
KEGG: Kyoto Encyclopedia of Genes and Genomes
RT-qPCR: Reverse transcription real-time quantitative PCR
NR4A1: Nuclear hormone receptor nuclear receptor subfamily 4, group a, member 1
KLF4: Kruppel-like factor 4
HSP70.2: Heat-shock protein 70.2
ZBTB16: Zinc finger and BTB domain containing 16
GAPDH: Glyceraldehyde-3-phosphate dehydrogenase
SE: Skippedexon
MXE: Mutually exclusive exon
A5SS: Alternative 5′ splice site
A3SS: Alternative 3′ splice site
RI: Retained intron
FPKM: Fragments per kilobase of exon model per million mapped fragments
HIF-1: Hypoxia inducible factor-1
ErbB: Erythroblastic leukemia viral oncogene homolog
PI3K-AKT: Phosphatidylinositol-3-kinase
TCA: Tricarboxylic acid cycle
cGMP-PKG: Cyclic guanosine monophosphate-protein kinase G
NAFLD: Nonalcoholic fatty liver disease
MAPK: Mitogen activated protein kinase
TGF-beta: Transforming growth factor beta
mTOR: Mechanistic target of rapamycin
cAMP: Cyclicadenosine monophosphate
ErbB: Erythroblastic oncogene B
