# Supplementary material for: Transcriptome profiling of longissimus dorsi during different prenatal stages to identify genes involved in intramuscular fat deposition in lean and obese pig breeds
Source: Mol Biol Rep. 2024 Mar 5;51(1):386. doi: 10.1007/s11033-023-09088-8 (PMC10914898; doi:10.1007/s11033-023-09088-8)
Supplement: Supplementary file 1 — Supplementary material 1 (DOCX 61.6 kb) [file 11033_2023_9088_MOESM1_ESM.docx]

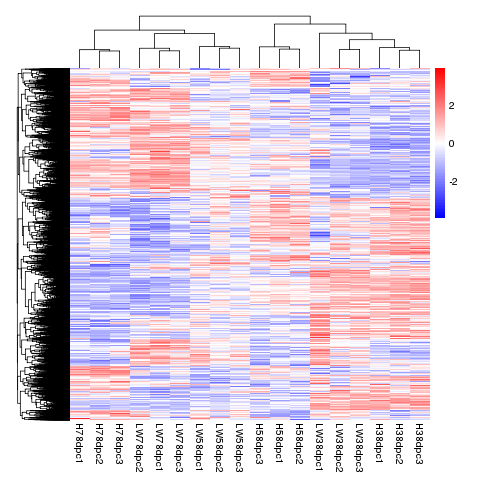


Supplement Fig.1 Cluster analysis of the DEGs between these two breeds. The color bars indicated the fold change of genes.
